# Pyogenic Splenic Abscess With Concurrent Active Plasmodium falciparum Malaria and Sickle Cell Trait: A Case Report

**DOI:** 10.7759/cureus.102272

**Published:** 2026-01-25

**Authors:** Shamon Gumbs, Winnie Gikunda, Herlyne Das, Jeremiah Douchee, Cameron A Wilkinson

**Affiliations:** 1 Department of Surgery, Columbia University College of Physicians and Surgeons, Harlem Hospital Center, New York, USA; 2 Department of Surgery, Woodhull Medical Center, Brooklyn, USA; 3 Department of Surgery, Columbia University College of Physicians and Surgeons, New York, USA

**Keywords:** malaria, malaria infection, spleen, splenic abscess, splenic aspiration

## Abstract

Splenic abscess is a rare but life-threatening condition with poorly understood links to malaria and hemoglobinopathies. We present the case of a young male from West Africa who developed a pyogenic splenic abscess with concurrent *Plasmodium falciparum *malaria and sickle cell trait. Clinical data were collected through retrospective chart review, and a literature review was conducted focusing on splenic abscess in the context of malaria and hemoglobinopathies. The patient presented with abdominal pain and fever after initiating antimalarial treatment. Imaging revealed a large splenic abscess. Percutaneous drainage yielded purulent fluid, and cultures identified *Fusobacterium *species. Laboratory findings confirmed sickle cell trait and active malaria. Treatment included antimalarial therapy and broad-spectrum antibiotics with anaerobic coverage. The patient recovered completely after drainage and antimicrobial therapy. This case highlights the importance of considering anaerobic bacterial pathogens, particularly *Fusobacterium *species, in splenic abscesses among patients from malaria-endemic regions. The concurrent presentation of bacterial infection, active malaria, and sickle cell trait represents a rare scenario demonstrating complex interplay between parasitic infection, hemoglobinopathies, and bacterial superinfection. Healthcare providers should maintain high suspicion for bacterial splenic infections in travelers with splenic complications, even when malaria is diagnosed.

## Introduction

Malaria remains one of the world's most significant infectious diseases, with an estimated 249 million cases and 608,000 deaths reported globally in 2022 [1]. The disease burden is concentrated in Sub-Saharan Africa, which accounts for approximately 95% of malaria cases and 96% of malaria-related deaths [1]. *Plasmodium falciparum*, the most dangerous of the five human malaria parasites, causes the majority of severe disease and mortality [2]. Malaria is transmitted through the bite of infected female Anopheles mosquitoes. Once in the human bloodstream, the parasites travel to the liver, where they multiply before re-entering the bloodstream and infecting red blood cells. This blood-stage infection causes the characteristic symptoms of malaria: fever, chills, headache, and body aches. In severe cases, complications can include cerebral malaria (brain involvement), severe anemia (low red blood cell count), acute respiratory distress syndrome (lung failure), and multi-organ dysfunction [2]. The spleen plays a critical role in the immune response to malaria by filtering parasitized red blood cells and mounting immune responses against the parasite [3].

Sickle cell trait (SCT), characterized by the inheritance of one normal hemoglobin gene (HbA) and one sickle hemoglobin gene (HbS), provides significant protection against severe *P. falciparum *malaria [4]. This genetic advantage has led to the persistence of the sickle cell gene in malaria-endemic regions, where SCT prevalence ranges from 10-40% in Sub-Saharan Africa [5]. The protective mechanisms of SCT against malaria are multifactorial and include enhanced clearance of parasitized red blood cells by the spleen, reduced parasite multiplication within red blood cells, and modulation of immune responses [4]. Individuals with SCT typically do not experience the severe complications associated with sickle cell disease (homozygous HbSS), as they possess sufficient normal hemoglobin to maintain adequate oxygen delivery under most circumstances. However, SCT is not entirely benign and has been associated with certain clinical complications, including splenic infarction, renal complications under conditions of extreme physical exertion or dehydration, and potential interactions with other disease processes [6].

Splenic abscess is a rare but serious condition with an estimated incidence of 0.05-0.7% in the general population [7]. The condition is more common in immunocompromised individuals, patients with hematologic disorders, those with endocarditis, and individuals who have experienced splenic trauma or infarction [7]. The most common causative organisms include aerobic bacteria such as *Staphylococcus aureus*, *Streptococcus species*, and *Escherichia coli*, as well as anaerobic bacteria and, less commonly, fungi [8]. Clinical presentation typically includes fever, left upper quadrant abdominal pain, and constitutional symptoms such as malaise and weight loss. Diagnosis is usually made through imaging studies, particularly contrast-enhanced computed tomography (CT), which reveals characteristic features of abscess formation [7]. Management strategies include antimicrobial therapy tailored to culture results and source control through percutaneous drainage or, in severe cases, splenectomy [9].

The relationship between malaria and splenic complications, including abscess formation, is complex and incompletely understood. While malaria commonly causes splenomegaly (enlarged spleen) and can lead to splenic infarction or rupture in severe cases, the development of splenic abscess in the context of malaria is rarely reported [10]. The mechanisms by which malaria might predispose to bacterial splenic infections include immune dysregulation, splenic congestion and microinfarction creating a nidus for bacterial seeding, and the general immunosuppressive effects of acute parasitic infection [3]. The co-occurrence of malaria, hemoglobinopathies such as SCT, and bacterial infections represents a particularly challenging clinical scenario that requires careful diagnostic evaluation and comprehensive management.

*Fusobacterium *species are anaerobic gram-negative bacilli that are part of the normal flora of the oral cavity, gastrointestinal tract, and female genital tract [11]. These organisms are increasingly recognized as important pathogens in a variety of invasive infections, including bacteremia, liver abscesses, and other deep-seated infections [12]. *Fusobacterium necrophorum *is particularly notorious for causing Lemierre's syndrome, a severe condition characterized by thrombophlebitis of the internal jugular vein following oropharyngeal infection. The pathogenicity of *Fusobacterium *species is attributed to several virulence factors, including lipopolysaccharide, leukotoxin, and the ability to form biofilms [11]. Infections with *Fusobacterium *species require antimicrobial therapy with agents that provide adequate anaerobic coverage, such as metronidazole, beta-lactam/beta-lactamase inhibitor combinations, or carbapenems [13].

This case report presents a rare clinical scenario involving the concurrent presence of a pyogenic splenic abscess caused by *Fusobacterium *species, active *P. falciparum *malaria, and sickle cell trait in a young adult male from West Africa. The case highlights the diagnostic challenges, management considerations, and potential pathophysiologic interactions between these conditions. We also review the relevant literature on splenic abscess in the context of malaria and hemoglobinopathies to provide a comprehensive understanding of this uncommon clinical presentation.

## Case presentation

A 23-year-old male with no significant past medical history presented to the emergency department with left-sided abdominal pain. This pain radiated to the back and was accompanied by recurring fevers, headaches, and constipation. The patient had recently emigrated from West Africa approximately 2 to 3 weeks before presentation. Notably, the individual had sought medical attention 11 days earlier, receiving a diagnosis of malaria and commencing treatment with atovaquone-proguanil. Upon physical examination, abdominal tenderness was observed in the left upper and lower quadrants and the epigastric region.

Additionally, left costovertebral tenderness was noted. Absence of jaundice, pallor, guarding, and rebound tenderness was documented. Labs were notable for an elevated white blood cell count, a low hemoglobin level, and an elevated platelet count, as shown in Table 1. The patient tested positive for sickle cell screening. Serological testing for HIV-1 and HIV-2 antigen/antibody yielded non-reactive results. Additionally, nutritional markers revealed elevated vitamin B12 levels, elevated ferritin, low total iron-binding capacity, low iron saturation, and low iron levels, as seen in Table 1.

**Table 1:** Laboratory values and reference ranges TIBC: total iron-binding capacity

Labs	Value	Reference range	Unit
WBC	12.9	4.5-11.0	x 10^9/L
Hemoglobin	10.4	12.0-16.0	g/dL
Platelet	719	150-400	x 10^9/L
Vitamin B12	1620	160-190	pg/mL
TIBC	174	255- 450	µg/dL
Iron saturation	14	25-35	%
Iron levels	25	80-180	µg/dL
Ferritin	1349	12-300	ng/mL

**Figure 1:**
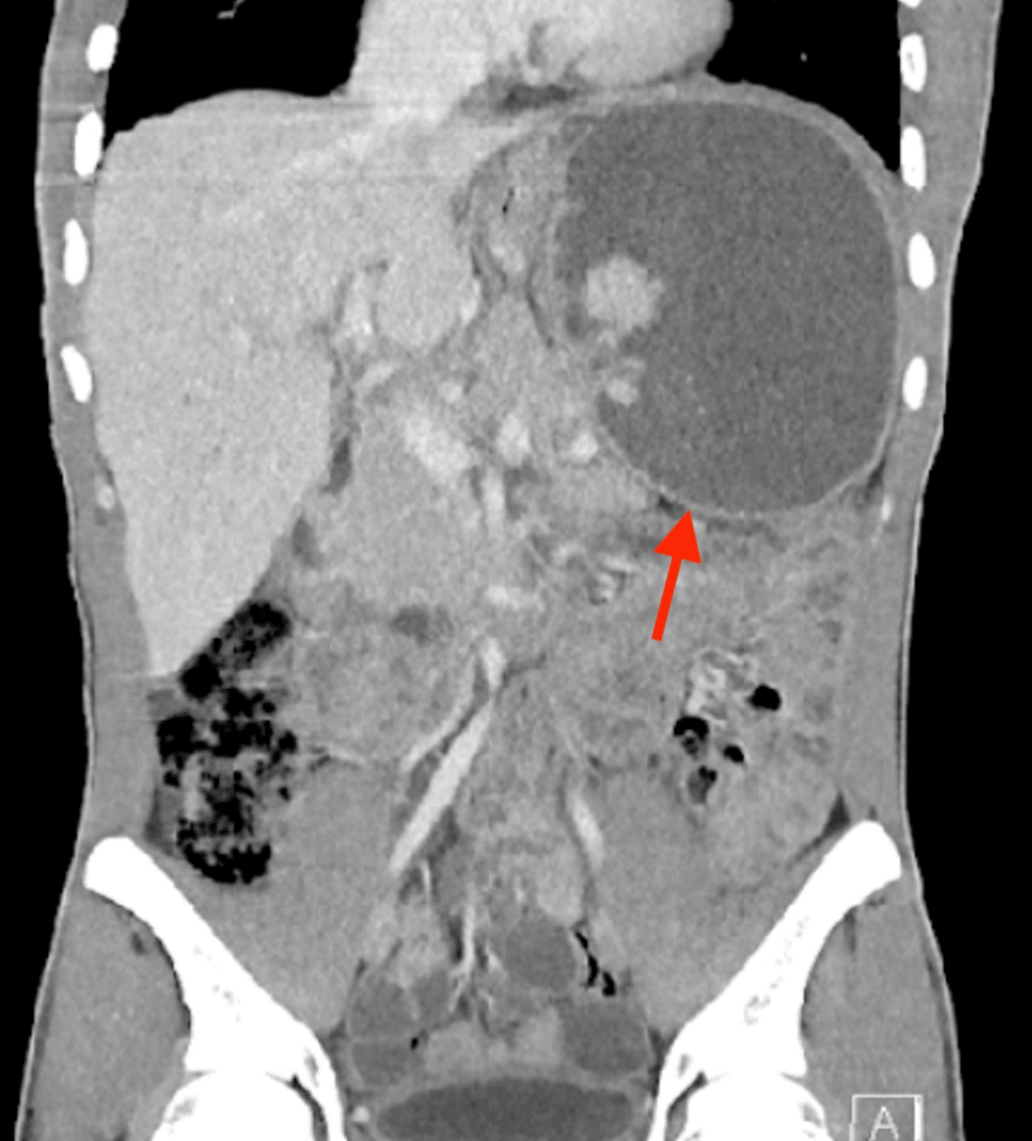
Coronal view CT abdomen/pelvis Coronal view CT abdomen/pelvis showing 12.7 x 7.8 x 14.5 cm heterogeneous lesion with areas of fluid attenuation more laterally in the left upper abdominal quadrant. Multiple hyperattenuating nodular lesions within the greater lesion, which may represent severely atrophic splenic tissue.

Hemoglobin electrophoresis, performed to evaluate the anemia and given the patient's African ancestry, revealed: HbS 39.6%, HbA 56.7%, HbA2 3.0%, and HbF 0.7%. These results are diagnostic of sickle cell trait (HbAS genotype). A computed tomography (CT) of the abdomen/pelvis was obtained and revealed a 12.7 x 7.8 x 14.5 cm heterogeneous lesion with areas of fluid attenuation laterally and increased attenuation medially. Nodular hyperattenuating lesions were observed within the larger lesion, accompanied by an enhancing rim (Figure 1). 

Interventional Radiology was consulted to drain the collection. CT-guided aspiration and drain placement were performed and yielded 1100 ml of bloody fluid with brown sediments, which was sent for further analysis. The cultures grew *Fusobacterium*, and serology was positive for *Entamoeba histolytica*. The patient was treated with atovaquone-proguanil, albendazole, metronidazole, and piperacillin-tazobactam. 

**Figure 2:**
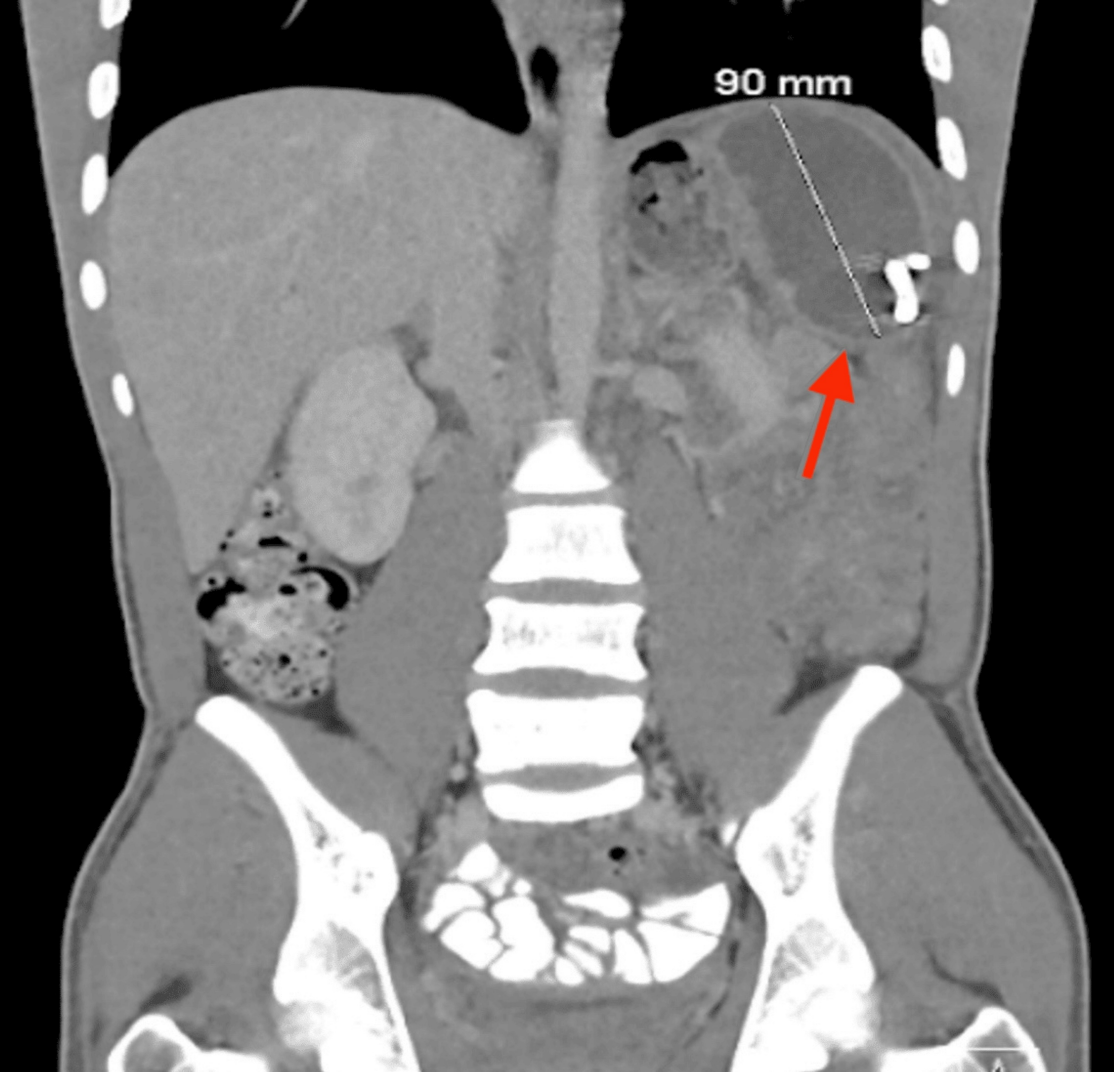
Coronal view CT abdomen/pelvis after percutaneous drainage Coronal view CT abdomen/pelvis showing a rim-enhancing heterogeneous lesion within the spleen, now measuring approximately 7.5 x 5.0 x 10.0 cm after percutaneous drainage.

A follow-up CT scan performed 5 days after drainage showed a significant reduction in abscess size, now measuring approximately 7.5 × 5.0 × 10.0 cm (Figure 2). The lesion maintained a rim-enhancing appearance with persistent heterogeneity, consistent with a resolving abscess. No new fluid collections were identified.

The patient showed rapid clinical improvement following drainage and initiation of antimicrobial therapy. The fever resolved within 48 hours, and the abdominal pain progressively decreased. He was discharged on hospital day 7 with oral antibiotics (amoxicillin-clavulanate and metronidazole for 14 days) and close outpatient follow-up. A repeat CT abdomen/pelvis was obtained 21 days post-drainage and showed the abscess measuring 3.4 x 3.2 x 4.1 cm. The peritoneal pigtail catheter was removed on day 16 post-discharge once the output was consistently <10 ml per day, and the patient was asymptomatic, without signs of systemic infection.

At the 4-week follow-up, the patient was completely asymptomatic, with resolution of abdominal pain and constitutional symptoms. Repeat laboratory studies showed normalization of white blood cell count and improvement in hemoglobin.

## Discussion

This case report describes a rare and complex clinical presentation involving the concurrent occurrence of a pyogenic splenic abscess caused by *Fusobacterium *species, active *Plasmodium falciparum *malaria, and sickle cell trait in a young adult male from West Africa. The case highlights several important clinical and pathophysiologic considerations regarding the interplay between parasitic infections, hemoglobinopathies, and bacterial superinfections.

Splenic abscess is an uncommon condition with an estimated incidence of 0.05-0.7% based on autopsy series and clinical reports [7]. The rarity of this condition is attributed to the spleen's robust blood supply, which typically prevents bacterial seeding, and its inherent antimicrobial properties mediated by resident macrophages and immune cells. However, certain predisposing factors significantly increase the risk of splenic abscess formation, including immunocompromised states (such as HIV infection, chemotherapy, or chronic corticosteroid use), hematologic disorders (including sickle cell disease, leukemia, and lymphoma), endocarditis with septic emboli, splenic trauma or infarction, and contiguous spread from adjacent infections [7], [8].

The microbiology of splenic abscesses has evolved, with recent case series showing a predominance of aerobic gram-positive cocci, particularly *Staphylococcus aureus *and *Streptococcus *species, followed by aerobic gram-negative bacilli such as *Escherichia coli *and *Klebsiella *species [8]. Anaerobic bacteria, including *Fusobacterium *species, *Bacteroides *species, and anaerobic streptococci, account for approximately 10-15% of cases [14]. Polymicrobial infections are reported in 5-10% of cases. The identification of *Fusobacterium *species in our patient is noteworthy, as this organism is an uncommon cause of splenic abscess and suggests possible hematogenous seeding from an oropharyngeal or gastrointestinal source [11], [12].

Understanding the role of malaria in this clinical scenario requires consideration of the spleen's central role in the host response to malaria infection. During the blood stage of malaria, the spleen filters parasitized red blood cells, removes intraerythrocytic parasites through a process called "pitting," and serves as a major site for the development of adaptive immune responses against the parasite [3]. Acute malaria infection typically causes splenomegaly due to increased blood flow, congestion, and cellular hyperplasia. In endemic areas where individuals experience repeated malaria infections, chronic splenomegaly (tropical splenomegaly syndrome or hyperreactive malarial splenomegaly) can develop [15]. While splenomegaly is common in malaria, other splenic complications are relatively rare.

Splenic rupture, though uncommon, is a well-recognized and potentially fatal complication of malaria, particularly in non-immune travelers with primary *P. falciparum *or *P. vivax *infection [16]. Splenic infarction has also been reported in malaria patients, likely resulting from microvascular occlusion by parasitized red blood cells and inflammatory thrombi. However, splenic abscess as a complication of malaria is exceedingly rare, with only scattered case reports in the literature [10], [17].

The mechanisms by which malaria might predispose to bacterial splenic abscess are not fully elucidated but likely involve multiple factors. First, malaria-induced splenomegaly and splenic congestion may create areas of relative ischemia and microinfarction, providing a nidus for bacterial seeding [3]. Second, acute malaria infection causes significant immune dysregulation, including transient immunosuppression characterized by impaired neutrophil function, altered cytokine profiles, and reduced T-cell responses, which may increase susceptibility to bacterial superinfections [18]. Third, the increased splenic blood flow and vascular permeability during acute malaria may facilitate hematogenous bacterial seeding of the spleen. Finally, malaria-associated hemolysis and anemia may lead to iron overload in the reticuloendothelial system, potentially promoting bacterial growth [19].

The presence of sickle cell trait in our patient adds another layer of complexity to this clinical presentation. Sickle cell trait (HbAS) is widely recognized for its protective effect against severe *P. falciparum *malaria, a selective advantage that has maintained the sickle cell gene at high frequencies in malaria-endemic regions [4, 5]. The mechanisms of protection are multifactorial and include enhanced splenic clearance of parasitized red blood cells (which undergo sickling under low-oxygen conditions), reduced parasite multiplication within HbAS red blood cells, impaired cytoadherence of parasitized cells to vascular endothelium, and modulation of immune responses [4].

Individuals with SCT typically have lower parasite densities and reduced risk of severe malaria complications such as cerebral malaria and severe anemia compared to individuals with normal hemoglobin (HbAA). However, SCT is not entirely benign and has been associated with certain clinical complications. Splenic infarction can occur in individuals with SCT under conditions of hypoxia, dehydration, or extreme physical exertion, particularly at high altitudes [6]. The proposed mechanism involves sickling of red blood cells within the splenic vasculature under low oxygen tension, leading to vascular occlusion and infarction. Splenic infarction, in turn, can predispose to bacterial superinfection and abscess formation due to tissue necrosis and impaired local immune function.

The relationship between SCT and susceptibility to bacterial infections is complex and not fully understood. Some studies have suggested that individuals with SCT may have subtle alterations in immune function, including changes in complement activation, cytokine production, and neutrophil function [20]. These immunologic differences, while generally not clinically significant in healthy individuals, may become relevant in the context of concurrent infections or other stressors. In our patient, the combination of SCT, active malaria infection, and possible splenic microinfarction may have created a perfect storm of conditions predisposing to bacterial abscess formation.

The identification of *Fusobacterium *species as the causative pathogen in this case warrants particular attention. *Fusobacterium *species are anaerobic gram-negative bacilli that are part of the normal flora of the oral cavity, gastrointestinal tract, and female genital tract [11]. Despite being commensals, these organisms are increasingly recognized as important opportunistic pathogens capable of causing serious invasive infections. *Fusobacterium necrophorum *is the most clinically significant species and is the causative agent of Lemierre's syndrome, a severe condition characterized by suppurative thrombophlebitis of the internal jugular vein following oropharyngeal infection, often with septic emboli to the lungs and other organs [12].

Other *Fusobacterium *species, including *F. nucleatum*, *F. varium*, and *F. mortiferum*, have been implicated in a variety of infections, including bacteremia, liver abscesses, brain abscesses, and intra-abdominal infections [11,12]. The pathogenicity of *Fusobacterium *species is attributed to several virulence factors, including lipopolysaccharide (endotoxin), leukotoxin (which can induce apoptosis of immune cells), hemagglutinin (facilitating adhesion to host cells), and the ability to form biofilms and co-aggregate with other bacteria [11].

The source of *Fusobacterium *bacteremia and disseminated infection is often the oropharynx or gastrointestinal tract, where these organisms are part of the normal flora. Disruption of mucosal barriers through dental procedures, periodontal disease, gastrointestinal inflammation, or other mucosal injuries can allow translocation of *Fusobacterium *into the bloodstream. In our patient, no obvious source of *Fusobacterium *bacteremia was identified, though subclinical periodontal disease or gastrointestinal translocation in the setting of malaria-induced gut inflammation are possible explanations. Treatment of *Fusobacterium *infections requires antimicrobial agents with good anaerobic coverage. Metronidazole is highly active against *Fusobacterium *species and is often considered the drug of choice for anaerobic infections [13].

Beta-lactam/beta-lactamase inhibitor combinations such as piperacillin-tazobactam and amoxicillin-clavulanate are also effective, as are carbapenems [13]. Clindamycin, historically used for anaerobic infections, has shown increasing resistance among *Fusobacterium* species in some regions and is no longer universally recommended as first-line therapy. Our patient received combination therapy with piperacillin-tazobactam and metronidazole, followed by oral amoxicillin-clavulanate and metronidazole, with excellent clinical response.

From a management perspective, the approach to splenic abscess requires a combination of antimicrobial therapy and source control. Empiric antimicrobial therapy should be initiated promptly upon diagnosis and should provide broad-spectrum coverage for aerobic and anaerobic bacteria pending culture results [9]. Common empiric regimens include piperacillin-tazobactam, carbapenems (such as meropenem or imipenem), or combination therapy with a third-generation cephalosporin plus metronidazole. Once culture results and susceptibility data are available, antimicrobial therapy should be tailored to the specific pathogen(s) identified. Source control is a critical component of splenic abscess management. Options for source control include percutaneous drainage (either catheter drainage or aspiration), splenectomy, or, in select cases, conservative management with antibiotics alone [9].

Percutaneous drainage has become the preferred initial approach for most splenic abscesses, as it is less invasive than splenectomy and preserves splenic function, which is important for long-term immunity against encapsulated bacteria [9]. Splenectomy is reserved for cases with multiple abscesses, failed percutaneous drainage, splenic rupture, or hemodynamic instability. Conservative management with antibiotics alone may be considered for small abscesses (<3 cm) in stable patients, though this approach requires close monitoring and has higher failure rates. The duration of antimicrobial therapy for splenic abscess is not well-standardized but typically ranges from 4-6 weeks, with initial intravenous therapy followed by transition to oral therapy once clinical improvement is demonstrated [9]. Follow-up imaging is important to document resolution of the abscess and to detect complications such as recurrence or splenic rupture.

In our patient, CT-guided percutaneous drainage yielded 1,100 mL of purulent material and provided both diagnostic information (culture identification of *Fusobacterium *species) and therapeutic benefit (source control). The patient was treated with a total of four weeks of antimicrobial therapy, with initial intravenous therapy followed by oral therapy, and showed complete resolution of the abscess on follow-up imaging.

An incidental finding in our patient was a markedly elevated vitamin B12 level of 1,847 pg/mL (reference range: 200-900 pg/mL). While vitamin B12 deficiency is a well-recognized clinical problem, elevated vitamin B12 levels are less commonly discussed but can be clinically significant. Elevated vitamin B12 levels have been associated with several conditions, including hematologic malignancies (particularly myeloproliferative disorders and acute leukemia), liver disease (due to release of B12 from hepatocytes), renal failure, and solid tumors [19]. Elevated B12 can also be seen in the context of acute inflammation and infection, possibly related to increased production of B12-binding proteins (transcobalamin and haptocorrin) as acute phase reactants.

In the context of our patient, the elevated B12 level was most likely related to the acute inflammatory state associated with the splenic abscess and malaria infection, rather than indicating an underlying hematologic malignancy or liver disease. The patient's liver function tests were only mildly elevated, and there were no other clinical or laboratory features suggestive of malignancy. The B12 level was not rechecked after resolution of the acute infection, but normalization would be expected if the elevation was indeed related to acute inflammation.

This case highlights several important clinical lessons for healthcare providers, particularly those practicing in non-endemic areas who may encounter travelers or migrants from malaria-endemic regions. First, splenic complications in malaria patients are not limited to splenomegaly and rupture; bacterial superinfections, including splenic abscess, should be considered in patients with persistent or worsening symptoms despite appropriate antimalarial therapy. Second, the presence of hemoglobinopathies such as sickle cell trait may increase the risk of splenic complications and should be evaluated in patients from endemic regions presenting with splenic pathology. Third, anaerobic bacteria, particularly *Fusobacterium *species, should be considered as potential pathogens in splenic abscesses, and empiric antimicrobial therapy should include anaerobic coverage.

Fourth, percutaneous drainage is an effective and spleen-preserving approach to managing splenic abscesses and should be considered as first-line therapy when feasible. The pathophysiologic interplay between malaria, sickle cell trait, and bacterial infection in this case remains incompletely understood and warrants further investigation. It is possible that malaria-induced splenic changes (congestion, microinfarction, immune dysregulation) created a favorable environment for bacterial seeding, and that the presence of sickle cell trait further contributed to splenic dysfunction and susceptibility to infection. Alternatively, the three conditions may have occurred coincidentally without direct causal relationships. Larger case series and mechanistic studies would be needed to clarify these relationships.

## Conclusions

This case report describes a rare and complex clinical scenario involving a large pyogenic splenic abscess caused by *Fusobacterium *species in a patient with concurrent active *Plasmodium falciparum *malaria and sickle cell trait. The temporal relationship between malaria diagnosis and abscess development, combined with the unusual causative organism, suggests a potential pathophysiological link between these conditions.

The case highlights several important clinical lessons: the need to maintain a broad differential diagnosis in patients from malaria-endemic regions presenting with abdominal pain and fever, the value of advanced imaging in diagnosing splenic pathology, the efficacy of percutaneous drainage combined with antimicrobial therapy as a spleen-preserving approach to splenic abscess management, and the importance of obtaining microbiological cultures to guide targeted therapy. The successful outcome in this case, with complete resolution of the abscess and preservation of splenic function, supports the use of conservative management strategies for splenic abscess when feasible. The concurrent presence of sickle cell trait raises intriguing questions about potential interactions between hemoglobinopathies, malaria, and susceptibility to bacterial infections, warranting further investigation.

Healthcare providers caring for patients from malaria-endemic regions should be aware of the potential for bacterial complications, including splenic abscess, and should consider anaerobic organisms such as *Fusobacterium *species in the differential diagnosis of splenic infections. This case contributes to the limited literature on splenic abscess in the context of malaria and hemoglobinopathies and may inform the management of similar cases in the future.
